# Machine Learning Applications for Opioid Use Management in Chronic Cancer Pain: A Systematic Scoping Review

**DOI:** 10.7759/cureus.106838

**Published:** 2026-04-11

**Authors:** Anastasia Zompola, Thalis Asimakopoulos, Christina Iosifidou, Dimitris Orfeas Monopatis, Fotis Braimakis, Irene C Kouroukli

**Affiliations:** 1 Biostatistics, National and Kapodistrian University of Athens School of Medicine, Athens, GRC; 2 Anesthesiology, National and Kapodistrian University of Athens School of Medicine, Athens, GRC; 3 Anesthesiology, European University Cyprus, Athens, GRC; 4 Anesthesiology, Hippocration General Hospital, Athens, GRC

**Keywords:** ai and machine learning, chronic cancer pain, opioid misuse, opioid use, systematic scoping review

## Abstract

Chronic pain remains a critical clinical issue worldwide, with adverse effects on the quality of life of oncology patients. Meanwhile, the overuse of opioids to treat or alleviate chronic cancer pain has contributed to a global opioid crisis. The increasing accessibility of high-quality clinical datasets and computational frameworks has promoted the use of machine learning (ML) techniques in clinical practice to manage opioid consumption. This review investigates the current bibliography referring to the role of applied ML techniques in opioid administration in patients with chronic cancer pain.

The objective of the current scoping review, according to population, intervention, comparison, and outcome (PICO) standards, was to evaluate the effectiveness of ML techniques in monitoring opioid consumption in patients with chronic cancer pain.

This review includes scientific journal papers published from 2010 to 2024 that use healthcare data from patients with chronic cancer pain, apply machine learning techniques, and may address the potential consequences of the misuse of opioids. A systematic literature search, following the Preferred Reporting Items for Systematic Reviews and Meta-Analyses (PRISMA) guidelines, was performed in PubMed and Google Scholar databases. Data extracted include the study’s goal, dataset used, cohort selected, types of ML models created, model evaluation metrics, and the details of the ML tools and techniques used to create the models.

After conducting the screening process, 50 articles were identified, but only four focused specifically on or included data of patients with chronic cancer pain where ML techniques were applied. The four included studies showed high performance (area under the curve {AUC}: >0.8) in predicting opioid adherence, misuse, and long-term use. Although generalizability remains limited due to small sample sizes and a lack of external validation, it sets distinct limits in applying these methods in clinical use.

After a thorough review of recent literature, ML models demonstrated promising accuracy in predicting opioid adherence, misuse, and long-term use among patients with chronic cancer pain. However, these findings are based on studies with limited sample sizes and a lack of external validation, which restricts their generalizability. Future research should focus specifically on populations with chronic cancer pain and expand predictive models to incorporate a combination of clinical, psychosocial, biometric, and genomic data. This approach may enable more accurate, personalized, and safer opioid management in oncology care.

## Introduction and background

The integration of artificial intelligence (AI) and machine learning (ML) techniques in healthcare has transformed the clinical approach to complex conditions, such as opioid use disorder (OUD). OUD is a multifactorial illness marked by extended opioid use and characterized by the loss of control of opioid use, which is continued despite harm [1,2]. It is a serious adverse event that can result despite the therapeutic use of opioids in the management of chronic cancer pain. Clinically, OUD may manifest as physical dependence, psychological dependence, or both, with the condition potentially progressing from dependence to addiction. Core clinical features include an overpowering desire to use opioids, the development of opioid tolerance, and the emergence of withdrawal symptoms upon opioid discontinuation.

ML is a subfield of artificial intelligence (AI) that involves algorithms capable of autonomously performing tasks while continuously improving their accuracy and performance through experience and exposure to data. ML algorithms can be applied to prediction, pattern recognition, or data classification, and various metrics are used to assess their effectiveness. There are four primary types of ML algorithms: supervised, semi-supervised, unsupervised, and reinforcement learning [3]. In supervised learning, the algorithm is trained using labeled data, allowing it to predict outputs for new, unseen input data. Semi-supervised learning functions similarly but integrates both labeled and unlabeled data to enhance learning efficiency. Unsupervised learning, on the other hand, relies solely on unlabeled data, requiring the model to identify patterns and correlations from the input data. Finally, reinforcement learning involves algorithms that optimize decision-making by mimicking the trial-and-error learning process to achieve the best possible outcomes [4,5]. In the clinical domain also, natural language processing (NLP), a specialized field of AI, is widely used to extract meaningful insights from clinical text [6].

A more advanced subset of ML is deep learning (DL), which is currently considered a core technology. DL uses multilayered neural networks, called deep neural networks, to simulate the complex decision-making power of the human brain [5,7]. There are different types of DL models, such as convolutional neural networks, deep reinforcement learning, and recurrent neural networks. On the whole, these different AI tools can identify patterns and extract useful insights from complex medical data, making them invaluable in the field of medical practice [8].

Globally, cancer is one of the most prevalent diseases, and one of its most frequent and disabling symptoms is pain. Cancer-related pain may arise from the primary tumor itself or metastases or as a side effect of treatment. Chronic cancer pain can significantly impact patients’ quality of life, leading to dysfunction and potentially increasing mortality rates [9]. While pain itself is not life-threatening, prolonged exposure can worsen overall health outcomes [9,10]. Worldwide, the primary treatment for moderate to severe cancer pain is the prescription of opioids. For mild to moderate pain, weak opioids such as codeine, hydrocodone, and tramadol are recommended. As pain becomes more severe, a combination of strong opioids and non-opioid analgesics is typically prescribed for more effective pain management [11].

Opioid use disorder (OUD) is a multifactorial condition influenced by both genetic and clinical factors, making it challenging for clinicians to accurately assess and interpret the effects of opioid medications using only patient history or conventional statistical approaches [2,12]. Additionally, the heterogeneity of the disease, shaped by individual genetic and clinical profiles, further complicates diagnosis and management. These complexities can be better addressed through the application of artificial intelligence (AI) tools in clinical practice. Integrating AI into healthcare holds excellent potential for improving either early disease diagnosis or early prevention, as AI-driven tools can recognize patterns and characteristics in disease and even surpass human diagnostic capabilities [8,13,14]. Hence, the application of ML in patients with OUD may be useful for classifying, managing, and predicting patient outcomes.

This scoping review explores the role of ML techniques in the identification, prediction, and management of OUD in patients with chronic cancer. Given the limited number of eligible studies, a scoping methodology approach was adopted to synthesize current evidence and identify existing research gaps. Although the primary focus was to investigate studies that use patients with chronic cancer as a population sample, studies involving broader populations that included patients with chronic cancer pain were also considered, in order to maximize the amount of relevant research to better characterize opioid consumption patterns and associated risks within this clinical context. Ultimately, this scoping review aims to highlight the potential of ML-driven tools to support clinical decision-making and improve patient outcomes in the management of OUD.

## Review

Methods

Protocol Registration

This review was registered in the International Prospective Register of Systematic Reviews (PROSPERO) (protocol number: CRD42025634367), an international registry for systematic reviews in health and social care.

Compliance With Ethics Guidelines

This article is a synthesis of previously conducted research and does not include any new experimental studies involving human participants conducted by the authors.

Literature Review Strategy

A systematic search was conducted in PubMed and Google Scholar to identify relevant studies published from January 2010 to December 2024 that investigated the application of machine learning (ML) techniques in detecting opioid use disorder (OUD) among patients with cancer with chronic pain. In PubMed, the search strategy employed the following combination of text words: (“OUD” OR “opioid use disorder” OR “opioid dependence”) AND (“machine learning” OR “artificial intelligence”). In Google Scholar, advanced searches were performed using keywords such as “machine learning in opioid consumption,” “ML in opioid use disorder,” and “AI and opioid dependence,” targeting keywords within the title and abstract fields. The extracted articles were then imported into Rayyan (Rayyan Systems Inc., Cambridge, MA), where two reviewers independently screened the titles and abstracts.

Inclusion and Exclusion Criteria

Specific criteria were defined for the selection of articles included in this systematic scoping review. Eligible studies involved human participants who were oncology patients experiencing chronic pain. The included studies were required to analyze clinical or genetic characteristics of patients using machine learning (ML) techniques to assess or control opioid consumption. Only original studies published in English between 2010 and 2025, and freely available in full text, were included.

Data Synthesis

This study used aggregated data where possible, in accordance with the Preferred Reporting Items for Systematic Reviews and Meta-Analyses (PRISMA) guidelines [15].

Given the small number of cancer-specific studies identified, quantitative synthesis techniques such as meta-analysis or meta-regression were not feasible.

Data Collection Process

The data gathered encompassed AI/ML methods employed, input variables and predicted outcomes, approaches used for model validation, and metrics to evaluate models’ performance. The overall quality of the included articles was assessed by focusing on appraising both the risk of bias (ROB) and adherence to reporting guidelines. Since studies focusing exclusively on patients with chronic cancer pain were limited, articles that included at least a portion of patients with chronic cancer pain in their study population were also considered. To assess the risk of bias, the Prediction model Risk Of Bias Assessment Tool (PROBAST) was utilized, which evaluates four key domains, participants, predictors, outcomes, and analysis, through 20 methodological questions to establish the overall risk of bias [16]. To additionally report the quality of the gathered articles, transparent reporting of a multivariable prediction model for individual prognosis was employed to examine whether the included studies reported all the necessary details for the theme of the review.

The scripts used for generating the graphs are publicly available on GitHub at https://github.com/alphazita.

Results

Search and Screening Results

**Figure 1:**
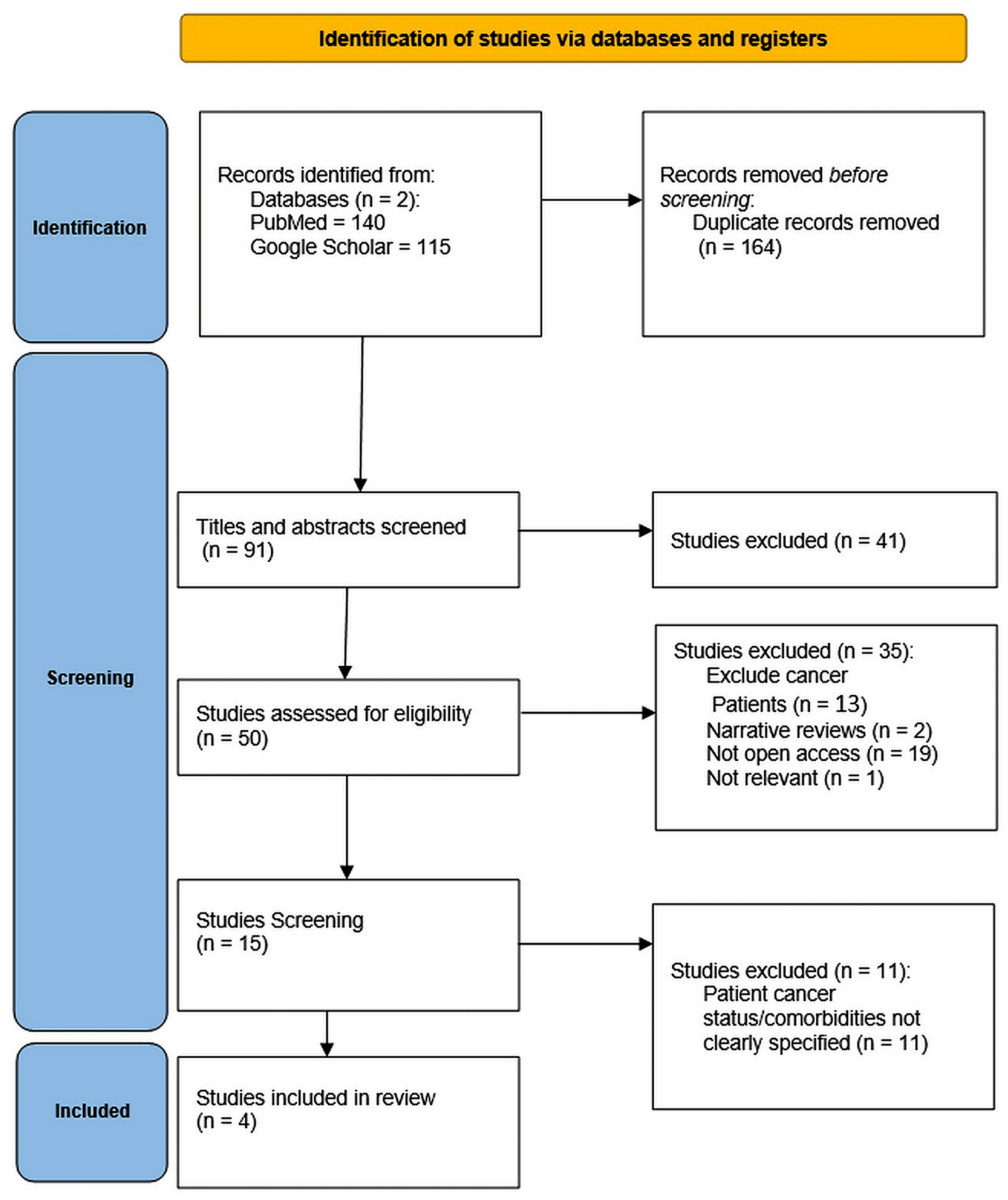
PRISMA Flowchart PRISMA: Preferred Reporting Items for Systematic Reviews and Meta-Analyses

In the initial screening, the search yielded a total of 255 published articles between 2010 and 2025. After importing the results into Rayyan, a web-based tool designed to support systematic reviews by enabling efficient de-duplication, blinded screening, and the resolution of conflicts among reviewers, 164 duplicate articles were identified and removed. A total of 91 unique articles remained for full screening. The remaining articles were then screened based on titles and abstracts, leading to 41 exclusions. Consequently, 50 articles were selected for full-text review to determine the final set of studies included. The search strategy process is illustrated in the following PRISMA flow diagram (Figure 1).

Study Characteristics

The primary aim of this review was to identify studies that applied machine learning techniques to analyze opioid use, misuse, or adherence specifically in patients with chronic cancer pain. However, only a limited number of studies focusing exclusively on the application of machine learning in populations with chronic cancer pain were found between 2010 and 2025 [17-20]. It was determined not to extend the search period, as the aim was to capture the most up-to-date literature. To maintain scientific relevance, the inclusion criteria were broadened to encompass studies involving mixed chronic pain conditions, provided that patients with cancer pain were included in the population. During the screening process, studies that explicitly excluded patients with chronic cancer pain were removed. Subsequently, all articles that had no implementation for patients with cancer were removed. Table 1 presents the final list of included studies, along with their population characteristics, applied ML methods, prediction targets, and main findings. A number of studies were excluded at this stage; although these studies involved populations with comorbidities and chronic pain, they did not include or specify whether some of the patients included in the study were suffering from cancer.

**Table 1:** Chronic Cancer Pain Articles and Mixed Ones Including Patients With Cancer ML, machine learning; AUC, area under the curve; SVM, support vector machine; MLP, multilayer perceptron; NLP, natural language processing; OUD, opioid use; PPV, positive predictive value; AUROC, area under the receiver operating characteristic; CUI, Controlled Unclassified Information

Author (Year)	Title	Country	Population/Data Source	Patients With Cancer	Sample Size	ML Method(s)	Outcome/Prediction Target	Main Results (Performance)
Held et al., 2024 [19]	Development and internal validation of a prediction model for long-term opioid use—an analysis of insurance claims data	Switzerland	Insurance claims (Helsana Group)	Mixed (includes 13% cancer)	266,476	Generalized linear regression and random forest	Long-term opioid use prediction	AUC, 0.927; accuracy, 81.6%
Liu et al., 2024 [18]	Opioid Nonadherence Risk Prediction of Patients with Cancer-Related Pain Based on Five Machine Learning Algorithms	China	Hospital-based cancer pain registry	Yes (cancer pain)	1,195	Logistic regression, random forest, XGBoost, SVM, and MLP	Opioid nonadherence prediction	AUC, 0.82; accuracy, 0.82
Poulsen et al., 2024 [17]	Developing a Framework to Infer Opioid Use Disorder Severity From Clinical Notes to Inform Natural Language Processing Methods: Characterization Study	USA	Electronic health record (HER) clinical notes (mixed: chronic pain, OUD, and control)	Mixed (includes chronic pain)	82 patients and 1,436 annotated sentences	NLP/annotation schema development	Characterization of OUD severity in clinical text	Inter-annotator agreement >70%; PPV ~0.71 for moderate/severe OUD detection
Sharma et al., 2020 [21]	Publicly available machine learning models for identifying opioid misuse from the clinical notes of hospitalized patients	USA	Adult hospitalized patients; electronic health record (EHR) clinical notes from a US health system (2007-2017)	Mixed (not cancer-specific)	1,000 inpatient encounters (case-control annotated dataset)	Convolutional neural network (CNN), max pooling network, and logistic regression; comparison with a rule-based model; NLP features including CUI codes, character-based and n-gram features	Identification/classification of opioid misuse from clinical notes	Best models achieved an AUROC of > 0.90; CNN and max pooling neural networks using CUI features

Risk-of-Bias Assessment and Quality of Studies

**Figure 2:**
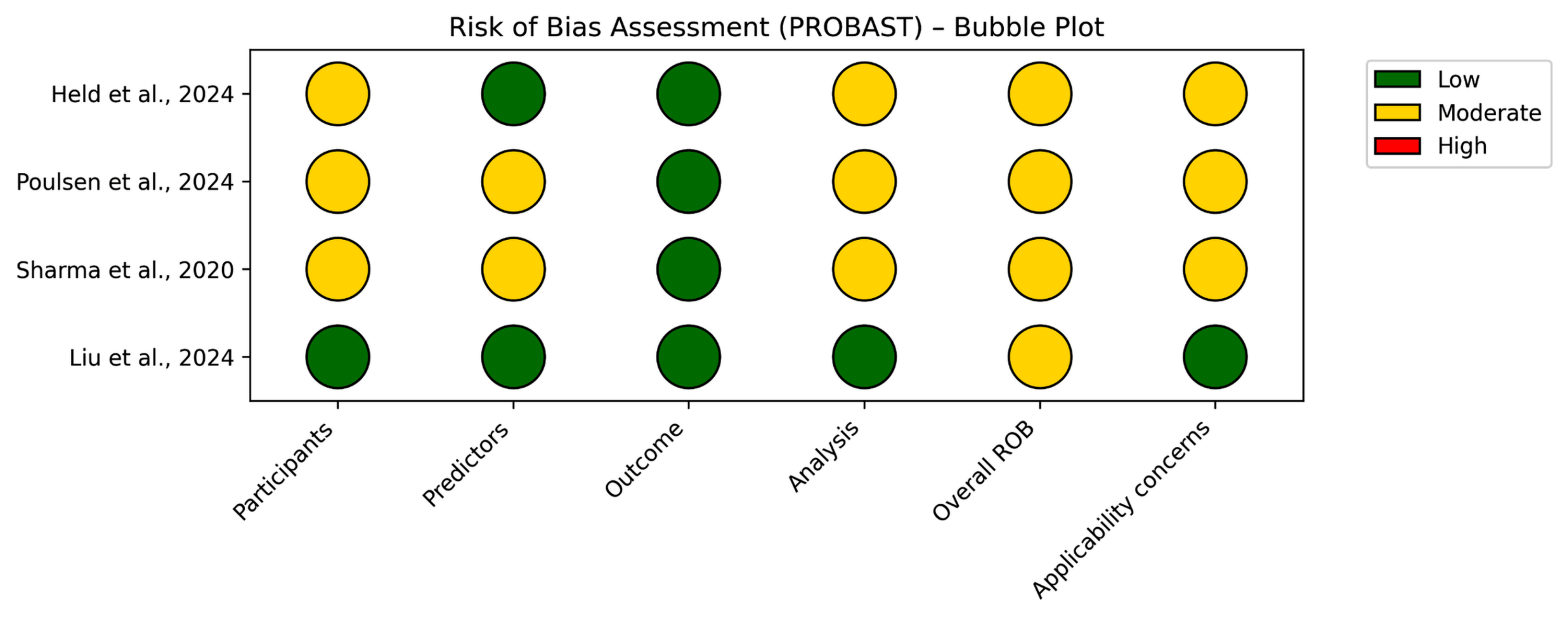
Risk of Bias (PROBAST) Source: Held et al., 2024 [19]; Poulsen et al., 2024 [17]; Sharma et al., 2020 [21]; Liu et al., 2024 [18] PROBAST, Prediction model Risk Of Bias Assessment Tool; ROB, risk of bias

In order to evaluate the risk of bias (ROB) in the final included studies, the PROBAST tool was applied. By applying this technique, a total of 20 signaling questions that focus on four domains (participants, predictors, outcome, and analysis) were answered. In Table 2, the results of the PROBAST application in the included studies are presented [16]. To quantify the relevance within each domain, a qualitative scale ranging from low to high relevance was applied (domain rated low/moderate/high/unclear) (Figure 2).

**Table 2:** Risk of Bias Score According to PROBAST PROBAST, Prediction model Risk Of Bias Assessment Tool; ROB, risk of bias; ML, machine learning; AUC, area under the curve; EHRs, electronic health records

Study (File Citation)	Participants	Predictors	Outcome	Analysis	Overall ROB	Applicability Concerns	Short Evaluation/Key Points
Held et al., 2024 [19]	Low → moderate	Low	Low	Moderate	Moderate	Some (mixed population, including 13% cancer)	Large insurance claim cohort (n ≈ 266k) → good. Predictors and outcome clearly defined (demographics, comorbidities, MED dose, etc.). Analysis: applied a multiple regression model and random forest analysis with validation and calibration reported (AUC, calibration plots, and Brier score) but lacking external validation; some transparency limits (data not publicly available and only a limited portion of the population, approximately 12% of the Swiss population) → moderate overall ROB
Poulsen et al., 2024 [17]	Moderate → high	Low → moderate	Low	Moderate	Moderate	Moderate (mixed cohorts; includes chronic pain but not cancer-specific)	Study develops/validates an annotation schema (27 classes) on clinical notes (82 patients and 1,436 sentences). Participants: small, selected cohorts (some groups, only European ancestry); may limit representativeness → moderate/high. Predictors (annotation classes) are well defined; outcome (severity score derived from annotations) is clearly specified; inter-annotator agreement acceptable (>70% in many batches). Analysis: appropriate for annotation study but small sample and limited external generalizability; study is not a predictive model per se (no ML model validation on external data). Overall ROB moderate
Sharma et al., 2020 [21]	Moderate	Low → moderate	Low	Moderate	Moderate	Moderate (mixed cohorts; includes chronic pain but not cancer-specific)	The cohort compares and evaluates the performance of three different algorithms: logistic regression, random forest, and gradient boosting. Participants: medical data from Tennessee Medicaid (n = 2.487). Predictors included demographics, medication use, comorbidities, and diagnostic codes extracted from EHRs. The data were appropriately defined, describing the samples, but feature handling (missing data and key feature selection) was not fully transparent. Analysis: Random forest achieved the best performance (AUC, 0.93; accuracy, 0.88). The validation was done only with internal data. Overall ROB moderate
Liu et al., 2024 [18]	Low → moderate	Low	Low	Low	Moderate	Low (specific to the population with cancer-related pain)	The study included 1,195 patients with chronic confirmed malignancies and moderate to severe cancer pain. The participants were recruited from a tertiary cancer center in China. Predictors included demographics and clinical predictors (43 variables, including pain characteristics, medication use, and psychosocial scores). The opioid nonadherence was based on both medical records and self-reported behaviors. Analysis: five ML algorithms (logistic regression, random forest, XGBoost, support vector machine, and multilayer perceptron) were compared to logistic regression, showing the best performance (AUC, 0.82; accuracy, 0.82). The validation was done only with internal data. Overall ROB was low to moderate

Based on the PROBAST, most of the included studies demonstrated a moderate overall risk of bias. The common limitation among the studies was the small sample sizes, according to the use of patients with chronic cancer pain. In addition, model validation was restricted to internal datasets, with no external or independent testing. This highlights the need for more focused validation in cancer-specific populations to strengthen the reliability of findings. The predictors and outcomes were generally well defined and appropriately applied in ML frameworks. Model performance was evaluated using standard metrics such as the area under the curve (AUC) and classification accuracy, supporting acceptable levels of predictive validity.

Findings by Theme

One of the primary constraints of the current investigation was identifying recently published research that only examines the impact of ML in opioid-dependent patients with chronic cancer pain. Due to the limited number of such specific studies, the inclusion criteria were broadened to incorporate research involving the population with chronic cancer pain that shares opioid use patterns, adherence issues, and risk behaviors. From the included studies, only one study specifically focused on patients with chronic cancer pain. The others involved populations with mixed chronic pain that may share comparable patterns of opioid use, adherence, and risk behavior. Therefore, insights from these models remain informative for understanding potential ML applications in oncology pain management. Performance metrics reported across studies were heterogeneous, with most studies presenting area under the curve (AUC), accuracy, sensitivity, and specificity. Confidence intervals and measures of uncertainty were rarely reported in the included studies.

ML Techniques in Opioid Prescription and Chronic Use

One thematic area included studies addressing long-term or chronic opioid use and ML models applied to identify risk factors associated with prolonged opioid therapy. Held et al. (2024) developed and internally validated a prediction model using insurance claim data, integrating demographic, clinical, and prescription-related predictors [19]. From the applied algorithms, both generalized linear regression and random forest models achieved strong performance (AUC = 0.93) and indicate that ML techniques can effectively identify patients at risk for long-term opioid dependence, including subgroups of patients suffering from chronic cancer pain. However, external validation and cancer-specific analyses limit the direct applicability of these findings to oncology in oncology-focused management.

ML in Opioid Misuse and Opioid Addiction Detection

Another research focus involved the use of ML in clinical or behavioral data to detect opioid misuse or OUD. Sharma et al. (2020) [21] and Poulsen et al. (2024) [17] applied NLP-based and deep learning models to electronic clinical notes to identify misuse behaviors and OUD severity levels. Both approaches achieved high internal validity (area under the receiver operating characteristic {AUROC} > 0.90), indicating that ML can effectively detect language or behavioral cues related to misuse [21,17]. Nonetheless, their populations included general hospitalized patients or patients with chronic pain and not specifically oncology patients with chronic pain. To strengthen their clinical relevance and expand the applicability of such models in exclusively cancer-related opioid management, external validation of such data is needed.

ML for Adherence and Response Prediction in Opioid Therapy in Cancer Pain

Liu et al. [18] examined opioid nonadherence specifically among patients with cancer pain by using five ML algorithms [6]. Demographic, psychosocial, and clinical features were used as predictors. The performance of the models was ideal. Logistic regression achieved the best performance, with an AUC of 0.82, an accuracy of 0.82, and a specificity of 0.71. The findings highlight the potential of ML to assess adherence behavior in the oncology pain population, offering valuable insight for personalized pain management. Nevertheless, the relatively small sample size limits generalizability, underscoring the need for larger samples and emphasizing the need for broader validation to allow the generalizability of the model.

Overall, in the freely available bibliography, there are articles examining misuse, adherence, and long-term opioid use. However, their translation to chronic cancer pain contexts remains limited by data heterogeneity, small sample sizes, and a lack of targeted validation.

**Figure 3:**
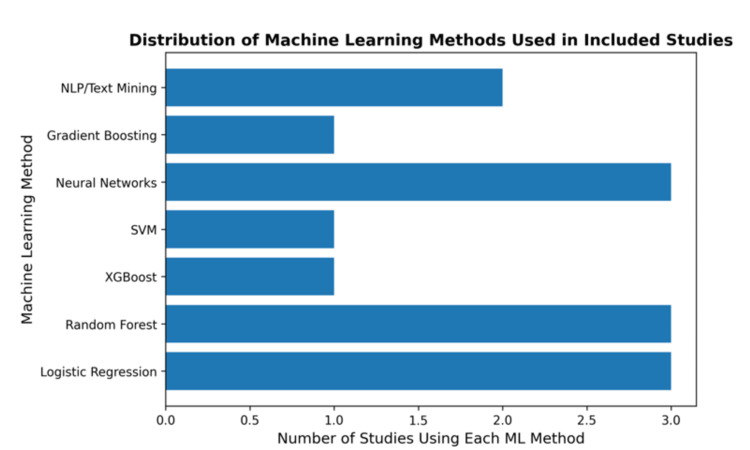
Distribution of Machine Learning Methods in the Included Studies ML, machine learning; SVM, support vector machine; NLP, natural language processing

Figure 3 shows the algorithm distribution among the different studies that were included in the review. ​

**Figure 4:**
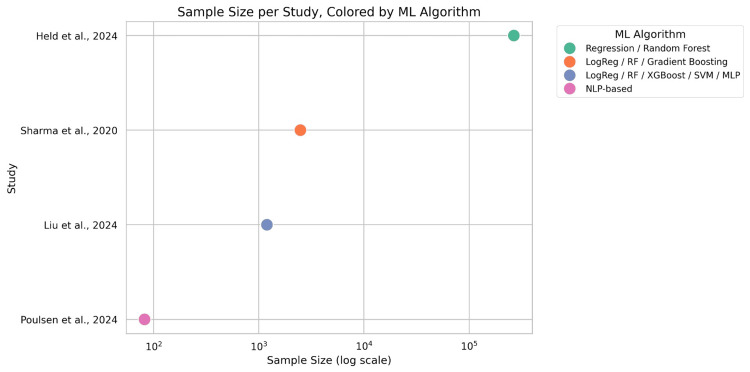
Sample Size per Study Source: Held et al., 2024 [19]; Poulsen et al., 2024 [17]; Sharma et al., 2020 [21]; Liu et al., 2024 [18] ML, machine learning; SVM, support vector machine; MLP, multilayer perceptron; NLP, natural language processing; RF, random forest

The variations in sample size among studies are presented in Figure 4. As there was distinct discrimination between the included studies according to the sample size, on the x-axis, the log value of each sample size is used. On the y-axis, the study is presented, and on the x-axis, the log per sample size in the graph also depicts the applied algorithms per study.

Discussion

After an extensive review of the recent literature, it was found that the application of machine learning (ML) methods in the use of opioids for patients with cancer with chronic conditions is still at a very early stage. Most models were used to predict adherence, misuse, or long-term opioid use, providing valuable insight into the potential use of ML in optimizing opioid therapy for oncology patients [17-19,21]. The algorithms used to analyze data in each study were mainly random forest, logistic regression, neural networks, and NLP. The average performance of the applied models was high, achieving an AUC of >0.8, but with internal validation only.

A targeted update of the literature (2024-2025) revealed that despite the increasing number of machine learning applications in opioid research, no newly published studies specifically address opioid management using ML in populations with chronic cancer pain.

Of the four studies included in this scoping review, only one directly evaluated ML-based prediction in a cancer-specific chronic pain cohort, Liu et al., who developed a model to predict opioid nonadherence in patients with cancer-related pain and reported strong performance metrics (AUC = 0.82) [18]. The remaining studies examined mixed populations with chronic pain, which may include oncology patients as subgroups, but were not designed to extract cancer-specific patterns [17,19,21]. This highlights a critical gap: while ML models show promise across broader chronic pain settings, datasets dedicated to the oncology population are necessary to develop clinically meaningful, cancer-centered opioid risk models. As an overall view, the findings suggest that ML approaches are technically feasible but clinically immature in oncology settings. For the gap in literature, it must be underlined that recent studies have primarily focused on acute cancer pain settings or on populations without cancer, such as opioid misuse prediction, overdose risk, and postoperative opioid use. This persistent gap underscores the lack of tailored, data-driven approaches for chronic cancer pain management and highlights an important direction for future research.

Cancer pain presents unique clinical characteristics that differentiate it from non-cancer chronic pain and influence the performance of ML-based opioid prediction systems. Cancer-related chronic pain is complex and has multiple clinical features that distinguish it from non-cancer chronic pain [22]. Its intensity may fluctuate due to tumor progression and treatment toxicities, making opioid prescribing more challenging. ML models must account for this vulnerability when supporting clinical decisions [14,23]. Studies also show that genetic predictors have not reliably estimated opioid requirements in patients with cancer, highlighting the difficulty of personalizing dosing [24]. Therefore, dedicated cancer-specific datasets are needed.

The integration of ML into clinical decision-making for opioid use is highly promising, particularly in light of the findings of the included studies, which demonstrated high performance in identifying patients at risk of opioid misuse, dependence, or nonadherence. Potential clinical applications include early warning systems for detecting adherence issues, optimized dosing recommendations, and personalized treatment strategies. However, for clinicians to make safe and effective decisions, it is essential that they fully understand and critically evaluate the models’ predictions. Moreover, the evaluation of these models must be based on cancer-specific clinical data to ensure relevance and reliability.

The current scoping review indicates that machine learning (ML) has the potential to improve current and future clinical practice. Specifically, the analyses conducted in each study led to different conclusions regarding adherence, misuse, and long-term opioid use. The studies conducted by Held et al. [19] and Liu et al. [18] suggest that opioid-related risk in patients with cancer is multifactorial and not driven by a single predictor. Regarding the NLP-based analyses performed by Poulsen et al. [17] and Sharma et al. [21], the way clinical information is documented plays an important role in model prediction. In particular, the findings of Sharma et al.’s study demonstrate that PHI-free modeling using standardized clinical concept representations can effectively capture relevant misuse patterns [21]. Taken together, these findings imply that early risk stratification and tailored clinical decision support are feasible, but they require rich cancer-specific features, careful feature selection, and attention to electronic health record documentation practices.

The key milestone of the present scoping review is emphasizing the need to expand the use of ML in patients suffering from chronic cancer pain. The findings of the included studies pinpoint the capability of the ML techniques to identify clinically relevant opioid-related outcomes such as nonadherence, misuse, and prolonged opioid use. Previous analysis on the application of ML in opioid research has primarily focused on populations with general pain or broader groups not specific to chronic cancer pain, highlighting the novelty and importance of focusing on cancer-related pain. From a clinical perspective, the utilization of ML in hospital practice could help personalize opioid therapy in oncology. It would allow therapy to be adjusted to each patient’s needs, improving monitoring and enabling the prevention or early detection of potential issues with opioid adherence. Another important advantage would be the use of these tools for real-time patient monitoring.

The main gaps and limitations identified in the included studies were related to small and heterogeneous datasets. The data often originated from insurance or hospital records, not specifically from patients with chronic cancer pain. Therefore, the datasets may have been limited or biased by their sources, with restricted or overly specific predictors. Future models should incorporate multimodal data, such as clinical, imaging, and genomic information, to make them more transparent, explainable, and suitable for clinical translation.

The limited number of studies identified in this review reflects the early stage of research on ML-based opioid management in chronic cancer pain. Machine learning applications in oncology pain have only recently begun to emerge, with few studies specifically targeted cancer-related chronic pain. A major barrier is the scarcity of high-quality clinical datasets that capture opioid consumption, pain trajectories, and treatment context in sufficient detail for predictive modeling [25]. Also, the high cost of the proper equipment to analyze these datasets imposes a lot of limitations [26]. Furthermore, most available databases do not integrate multimodal information such as genomics, patient-reported outcomes, and longitudinal treatment records, which limits model performance and generalizability [27]. As a result, most recently published cohorts apply ML techniques in broader populations with chronic pain, which may not fully represent the complexity of cancer-related chronic pain.

In addition to technical limitations, the use of ML in opioid management for cancer pain raises important ethical considerations. Many ML models operate as “black box” systems, providing predictions without transparent clinical reasoning. This can undermine clinician trust and limit their use in patient care [28]. Furthermore, models trained on limited or unrepresentative datasets may lead to biased clinical decisions, particularly for vulnerable populations with cancer [29]. Protecting patient privacy and autonomy is also critical, as oncology datasets often contain highly sensitive information that requires strong data security measures and compliance with health data regulations [30]. Addressing these ethical concerns is essential for the safe clinical deployment of ML-based opioid decision systems in oncology.

Future studies should focus on developing ML models that are trained and validated in chronic cancer pain cohorts. Comparing different ML architectures and combining reinforcement learning with deep learning could provide valuable insights into the mechanisms underlying chronic cancer pain and improve model interpretability. To make these tools clinically useful, it is necessary to validate them on external datasets and evaluate their performance using various metrics.

ML techniques are indeed very promising for opioid management in chronic cancer pain. However, their clinical translation requires robust, cancer-specific validation. The integration of ML into clinical practice represents a valuable step forward for clinicians, provided it is accompanied by collaboration between data scientists and pain specialists to enhance pain management in patients with chronic cancer pain.

Limitations

While the primary scope of the review was to study the application of ML techniques in opioid use in patients with chronic cancer pain, there were notable limitations in the available literature. To address this limitation, studies involving patients with cancer pain as a comorbidity were also included. The final included studies were characterized by heterogeneity in their populations and applied methods, making direct comparisons somewhat difficult. The sample size per study was also limited (<1,000 participants), reducing model robustness and generalizability to oncology settings. One commonality among the different studies was the lack of external validation. The metrics used to evaluate the performance of each model did not include calibration, precision-recall, or F1 scores, making it difficult to assess the reliability of the models. Almost none of the studies performed external testing on independent datasets. Most studies used customized data for both training and validations, such as insurance records or hospital data, tailored to the specific needs of each study, thereby reducing the generalizability of the models and posing a risk of bias.

There is no doubt that the application of AI techniques in pain management can greatly enhance patients’ quality of life. However, there are specific limitations that must be overcome to maximize the benefits of AI for both patients and healthcare providers. The main obstacles relate to the operational nature of AI tools, which often function as “black boxes” [28]. There is a need for greater transparency; both patients and clinicians should understand how models make decisions, what data are used, and what assumptions underlie their predictions. Models can also exhibit bias, often due to a lack of diversity or insufficient training data for certain patient groups. Another challenge concerns the inherent complexity of pain assessment. Accurately recognizing pain is essential for effective therapy but remains difficult because of its subjective character and multiple influencing factors, including emotional, behavioral, and lifestyle components [22,31]. Finally, it is crucial to maintain data privacy. Given that patients’ information is vulnerable, any AI tool must safeguard confidentiality, prevent discrimination, and strengthen rather than replace the patient-clinician relationship.

Directions

It is true that the use of machine learning (ML) algorithms for monitoring opioid consumption in patients with chronic cancer pain is highly promising. To address the current limitations, several steps can be taken. Given the unique clinical and biological features of cancer pain, future ML models should be trained and evaluated in dedicated cancer-specific datasets that incorporate the different characteristics such as pain severity, cancer stage, treatment context (e.g., palliative), and genetic markers associated with opioid metabolism [12,32]. Cancer pain is heterogeneous, and models developed in broader populations with chronic pain may not capture the different expression of specific features, leading to decreased performance and limited generalizability across oncology patients.

Furthermore, it is essential to validate the performance of existing models using external datasets that are specifically representative of populations with chronic cancer pain, ensuring that their predictive accuracy remains consistent. In addition, model performance should be evaluated using a broader range of metrics, particularly those assessing generalizability, calibration, and potential over-fitting (e.g., Brier score, calibration slope, and F1 score). Creating strong evidence for the usefulness of ML in clinical settings will boost the integration of these technologies in clinical decision-making [33].

Expanding the datasets to include a combination of biometric, genetic, and clinical information would also be beneficial. Multimodal data offer deeper clinical insight. Also, integrating diverse predictors that provide a more comprehensive view of the patient could enable clinicians to make more accurate, individualized decisions, taking into account both therapeutic responses and potential adverse effects. The combination of different data types and the inclusion of patients with more chronic cancer pain reflects the multifactorial nature of chronic cancer pain.

## Conclusions

The present scoping review highlights that the implementation of machine learning techniques in monitoring opioid use among patients with chronic cancer pain remains at an early stage. Although the included studies demonstrated strong internal performance in different fields of application in opioid use, such as predicting opioid adherence, misuse, and long-term use, most were conducted in mixed or non-cancer-specific populations, limiting their direct applicability to oncology care. The findings suggest that ML can be applied in clinical decision-making as they achieved high performance in identifying patients at risk for opioid misuse or inadequate pain control, contributing to more personalized and safer pain management. However, the generalizability of the models is restricted by small sample sizes, limited external validation, and heterogeneous datasets. Future research should focus on heterogeneous, prospective studies including chronic cancer pain cohorts. To expand the efficiency of the model, integrating diverse data types such as clinical, psychosocial, biometric, and genomic information would be advantageous. Ultimately, ML tools hold significant promise in optimizing opioid use and improving the quality of life for patients with cancer experiencing chronic pain.
